# Supplementary material for: Structural Basis of the Interaction of the G Proteins, Gαi1, Gβ1γ2 and Gαi1β1γ2, with Membrane Microdomains and Their Relationship to Cell Localization and Activity
Source: Biomedicines. 2023 Feb 14;11(2):557. doi: 10.3390/biomedicines11020557 (PMC9953545; doi:10.3390/biomedicines11020557)
Supplement: Supplementary file 1 [file biomedicines-11-00557-s001.zip › biomedicines-2160222-supplementary.pdf]

biomedicines

MDPI

Article

Structural basis of the interaction of G proteins, $G\alpha i_1$, $G\beta_1\gamma_2$, and $G\alpha i_1\beta_1\gamma_2$, with membrane microdomains and their relationship to cell signaling, pathophysiology and drug discovery

Rafael Álvarez[1] and Pablo V. Escribá[1,*]

Supplementary Materials: The folowing supporting information has been provided: **Figure S1.** Example of immunoblotting analysis of mutant $G\gamma_2$ subunits overexpressed in Sf9 cells. Left panel, Immunoblotting showing expression of K64G $G\gamma_2$ subunit expressed in Sf9 cells, which were infected with Baculovirus containing the G protein subunit cDNA (I, infected) or cells which were not infected with it (C, Control). Right panel, Immunoblotting showing the expression of the double $G\gamma_2$ subunit mutants C68S-K64G and C68S-K65G (I) and their corresponding controls (C). **Figure S2.** Purification of WT and mutant $G\beta_1\gamma_2$ dimers overexpressed in Sf9 cells. The different G protein dimers were purified as described in the Materials and Methods section. Those liquid chromatography fractions rich in each mutant are shown with an asterisk after analysis on 10% polyacrylamide SDS-PAGE gels. **Figure S3.** Analysis of purified $G\beta_1\gamma_2$ dimers on nondenaturing gels. Proteins were fractioned on non-denaturing 8% polyacrylamide gels and detected with an anti- $G\gamma_2$ antibody (upper panel) or anti- $G\beta_1$ antibody (lower panel). R62: R62G; K64: K64G; K65: K65G; C68: C68S. **Figure S4.** Binding of $G\beta_1$ and $G\gamma_2$ monomers to Sf9 cell membranes. The graphs show the membrane-to-cytosol distribution of these G protein subunits, which were expressed independently in Sf9 cells. A, The $G\gamma_2$ wild-type and K64G subunits show a membrane (P, pellet) preference, whereas loss of the geranylgeranyl moiety (C68) induces a dramatic increase in the presence of this protein in the cytosol (SN, supernatant). B, The $G\beta_1$ subunit showed a preference for membranes. No $G\gamma_2$ subunit mutants were used in the present study. P: pellet; SN: supernatant. **Figure S5.** $G\beta_1\gamma_2$ dimer binding to Sf9 cell membranes. Distribution of wild-type (solid bars) and mutant (open bars) $G\beta_1\gamma_2$ heterodimers to Sf9 membranes (P, pellet) and soluble (SN, supernatant) fractions as determined with the anti-$G\gamma_2$ (A) and -$G\beta_1$ (B) antibodies. These two G protein subunits were co-expressed in Sf9 cells. The observed differences were most likely due to differences in antibody affinities and/or differential expression of these proteins in insect cells. These differences were reduced by the subsequent chromatographic purification process carried out before model-membrane binding experiments. **Figure S6.** Analysis of purified $G\alpha_1\beta_1\gamma_2$ trimers on nondenaturing gels. Proteins were fractioned on non-denaturing 8% polyacrylamide gels and detected with an anti- $G\alpha i_1$ antibody (A) or anti-$G\beta_1$ antibody (B). Panel C shows the recovery of different G protein heterotrimers after affinity chromatography. For other details, see text. C68: C68S; RKK: R62G-K64G-K65G. **Figure S7.** Effect of the $G\beta_1\gamma_2$ dimer on the binding of wild type and mutant $G\alpha i_1\beta_1\gamma_2$ trimers to Sf9 membranes. The binding of the wild-type and mutated $G\alpha i_1$ subunit was measured in Sf9 cell membranes in the presence (due to co-expression) or absence of wild-type $G\beta_1\gamma_2$ dimers. A, Effect of the $G\beta_1\gamma_2$-dimer on the binding of $G\alpha i_1$ subunits to Sf9 cell membranes as a function of the amount of $G\gamma_2$ subunit measured. B, Effect of the $G\beta_1\gamma_2$-dimer on the binding of $G\alpha i_1$ subunits to Sf9 cell membranes as a function of the amount of $G\beta_1$ subunit measured. C, Effect of the $G\beta_1\gamma_2$-dimer on the binding of non-myristoylated (Myr-$G\alpha$), non-palmitoylated (Pal-$G\alpha$) or diacylated (myristoylated and palmitoylated, WT $G\alpha$) $G\alpha_1$ protein with respect to the binding of the $G\alpha_1$ protein alone. **Figure S8.** Effect of $G\alpha_1$ mutations on the binding of $G\alpha i_1\beta_1\gamma_2$ heterotrimers to Sf9 membranes. The levels of the wild-type and mutated $G\alpha i_1$ subunits were measured in Sf9 cell membranes (P, pellet) and cytosol (SN, supernatant) from cells that co-expressed the wild-type $G\beta_1\gamma_2$ heterodimer. Data represent mean ± S.E.M. values; *$p<0.05$ with respect to WT $G\alpha i_1$; **$p<0.01$ with respect to WT $G\alpha i_1$; #$p<0.05$ between Myr- (G2A mutant) $G\alpha i_1$ and Pal- $G\alpha i_1$. **Figure S9.** Effect of mutations on $G\gamma_2$ on the binding of $G\alpha i_1\beta_1\gamma_2$ heterotrimers to Sf9 membranes. The levels of G protein heterotrimers with the wild-type and mutated $G\gamma_2$ subunits were measured in Sf9 cell membranes (P, pellet) and cytosol (SN, supernatant) from cells that co-expressed the alpha, beta and gamma

subunits indicated. Data represent mean ± S.E.M. values: *$p<0.05$, ***$p<0.001$ with respect to WT Gγ_2; ϕ$p<0.05$ between Gγ_2 C68S K64G and Gγ_2 C68S K65G; #$p<0.05$ between Gγ_2 C68S and Gγ_2 C68S K64G.

Figure S10. Representative immunoblots of G protein-membrane binding experiments. Wild type and mutant G proteins were incubated with preformed model membranes (liposomes) containing the different lipids indicated in the materials and methods section. Bound and free G proteins were separated by ultracentrifugation and samples from the pellet and supernatant were fractioned by electrophoresis (SDS-PAGE). G protein binding to membranes was quantified by immunoblotting using specific antibodies and known amounts of a G protein standard.

Supplementary material

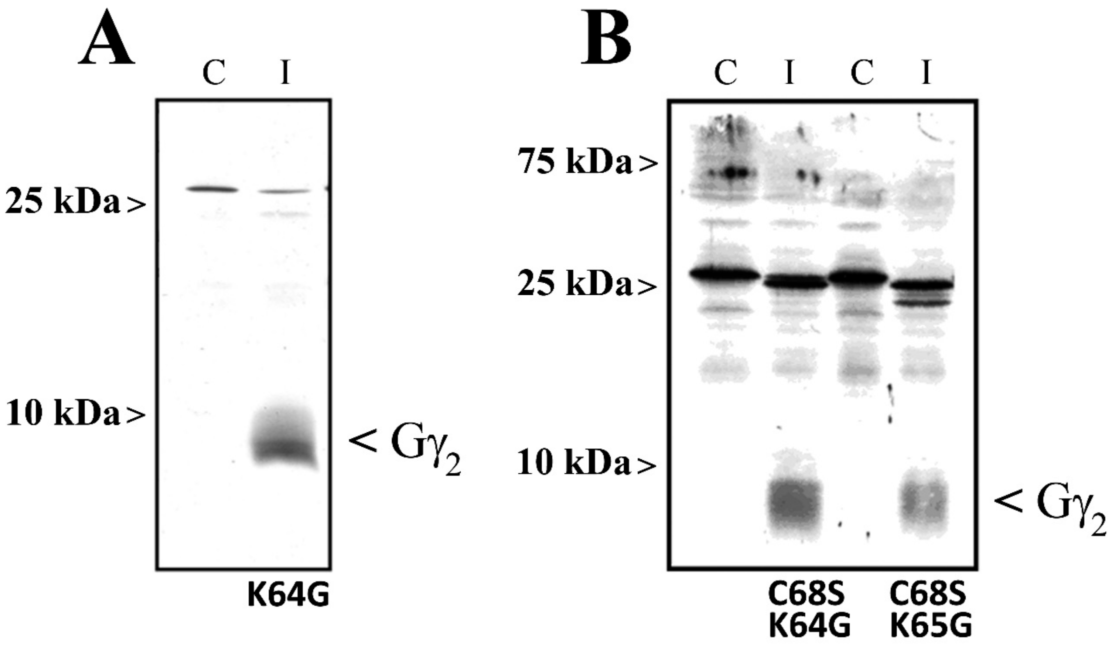

Figure S1

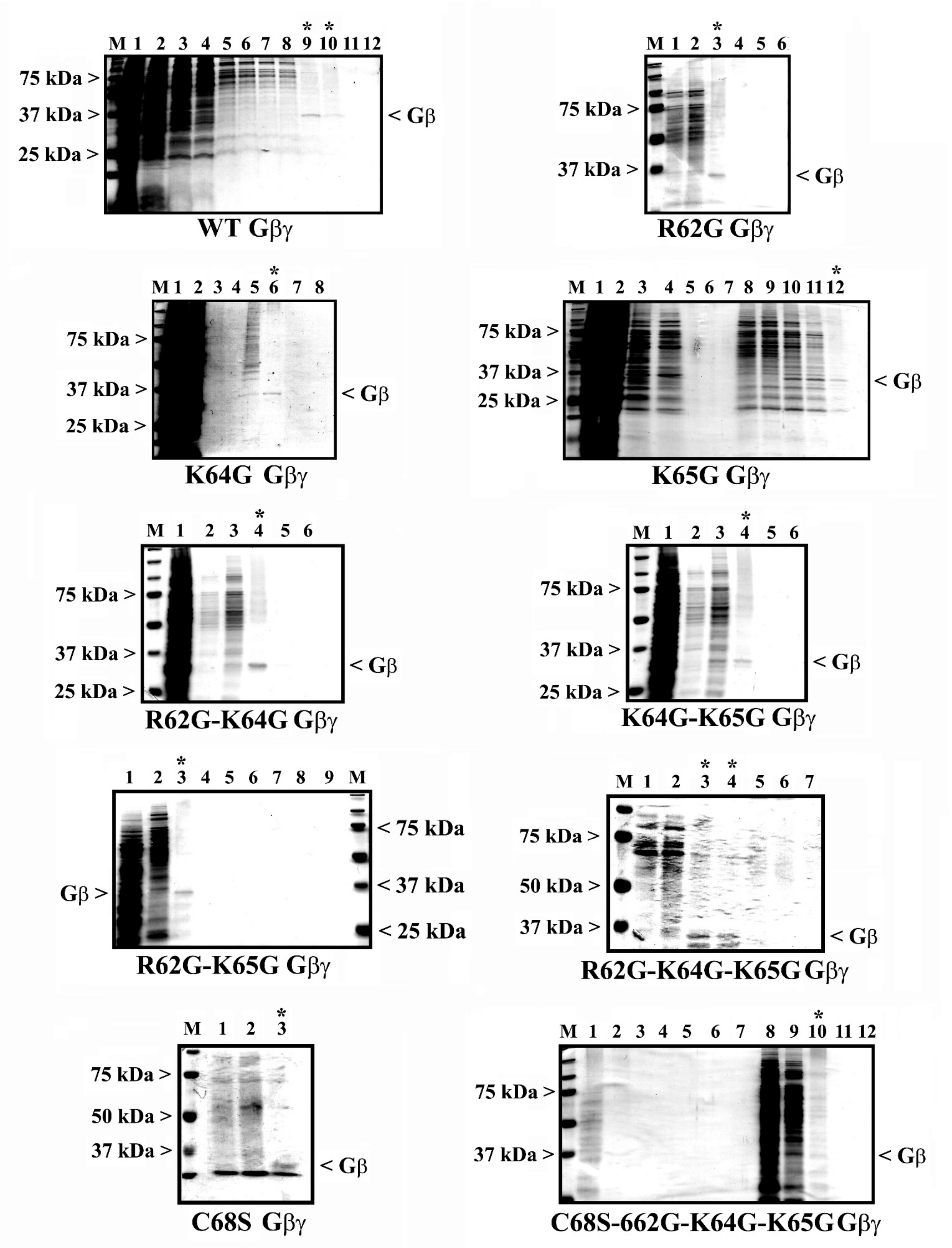

M 1 2 3 4 5 6 7 8 9 10 11 12
75 kDa >
37 kDa >
25 kDa >
< Gβ
WT Gβγ
M 1 2 3 4 5 6
75 kDa >
37 kDa >
< Gβ
R62G Gβγ
M 1 2 3 4 5 6 7 8
75 kDa >
37 kDa >
25 kDa >
< Gβ
K64G Gβγ
M 1 2 3 4 5 6 7 8 9 10 11 12
75 kDa >
37 kDa >
25 kDa >
< Gβ
K65G Gβγ
M 1 2 3 4 5 6
75 kDa >
37 kDa >
25 kDa >
< Gβ
R62G-K64G Gβγ
M 1 2 3 4 5 6
75 kDa >
37 kDa >
25 kDa >
< Gβ
K64G-K65G Gβγ
1 2 3 4 5 6 7 8 9 M
Gβ >
< 75 kDa
< 37 kDa
< 25 kDa
R62G-K65G Gβγ
M 1 2 3 4 5 6 7
75 kDa >
50 kDa >
37 kDa >
< Gβ
R62G-K64G-K65G Gβγ
M 1 2 3
75 kDa >
50 kDa >
37 kDa >
< Gβ
C68S Gβγ
M 1 2 3 4 5 6 7 8 9 10 11 12
75 kDa >
37 kDa >
< Gβ
C68S-662G-K64G-K65G Gβγ

Figure S2

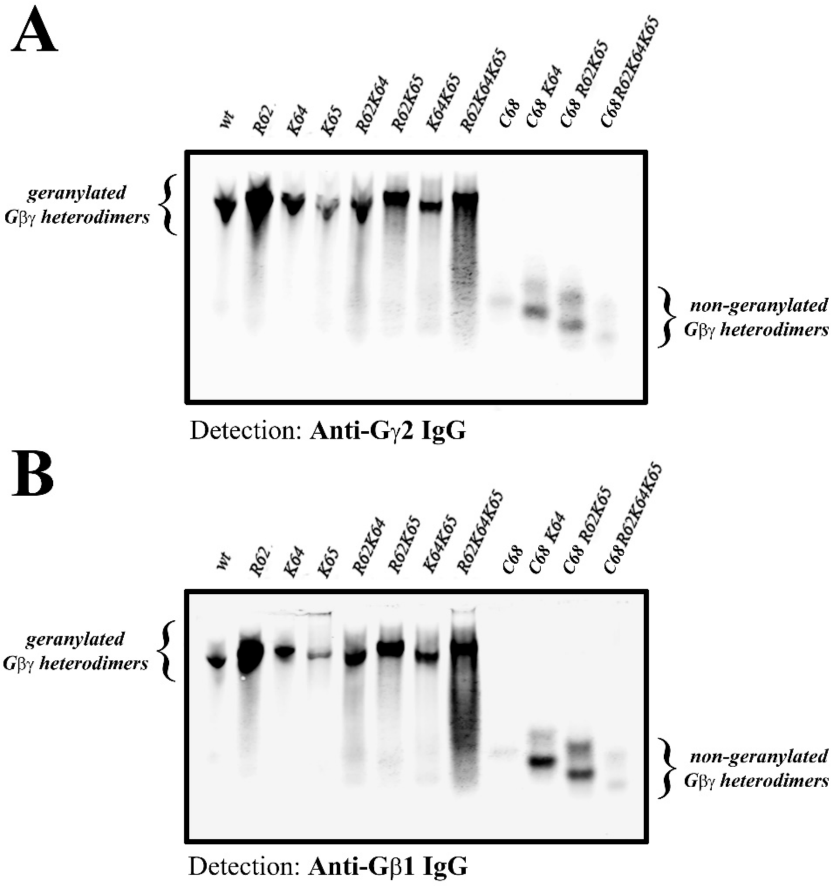
A
wt
R62
K64
K65
R62K64
R62K65
K64K65
R62K64K65
C68
C68 K64
C68 R62K65
C68R62K64K65
geranylated
Gβγ heterodimers
non-geranylated
Gβγ heterodimers
Detection: Anti-Gγ2 IgG
B
wt
R62
K64
K65
R62K64
R62K65
K64K65
R62K64K65
C68
C68 K64
C68 R62K65
C68R62K64K65
geranylated
Gβγ heterodimers
non-geranylated
Gβγ heterodimers
Detection: Anti-Gβ1 IgG

Figure S3

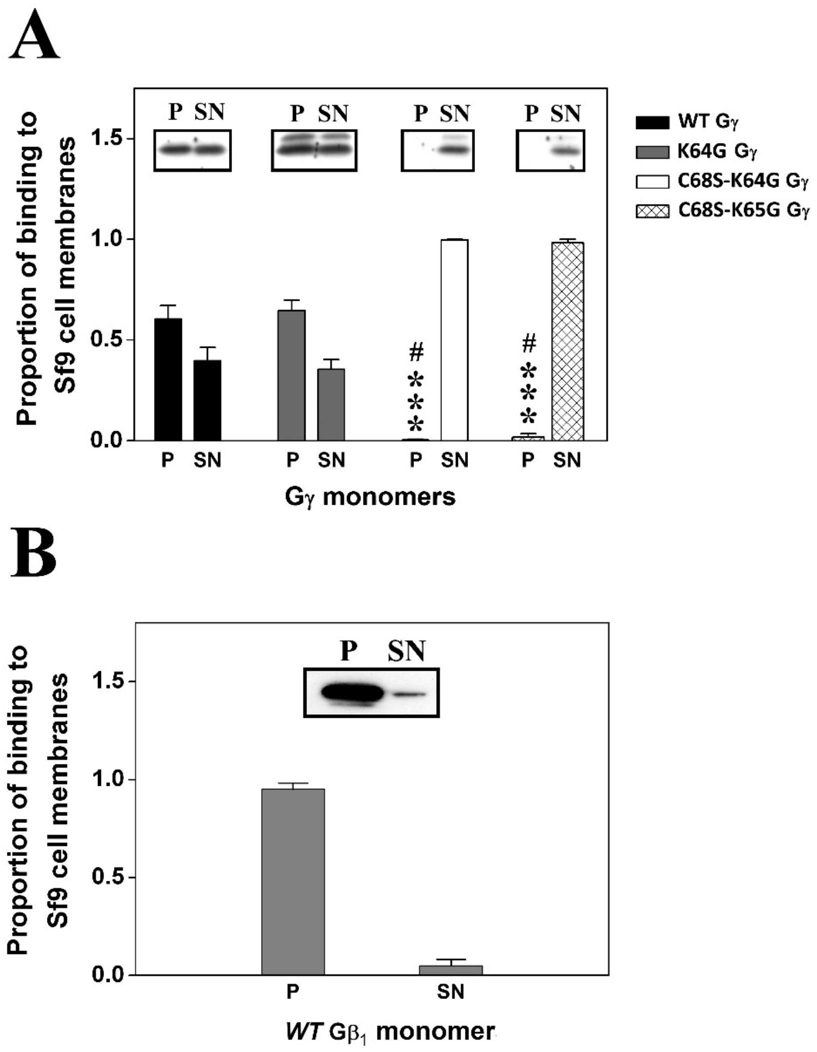
A
P SN
P SN
P SN
P SN
WT Gγ
K64G Gγ
C68S-K64G Gγ
C68S-K65G Gγ
Proportion of binding to Sf9 cell membranes
1.5
1.0
0.5
0.0
#
***
P SN P SN P SN P SN
Gγ monomers
B
P SN
WT Gβ1 monomer

Figure S4

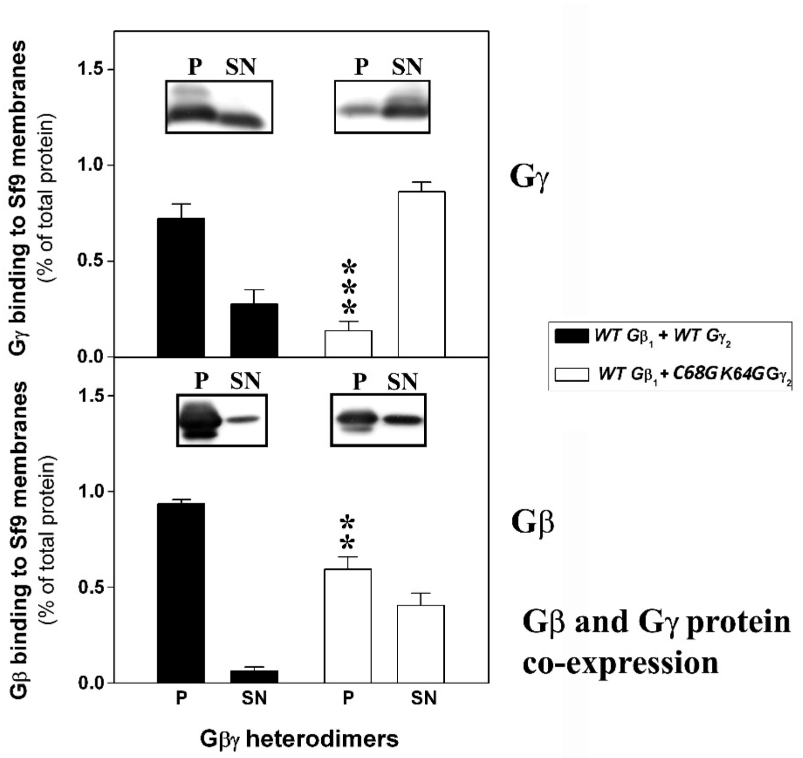

Figure S5

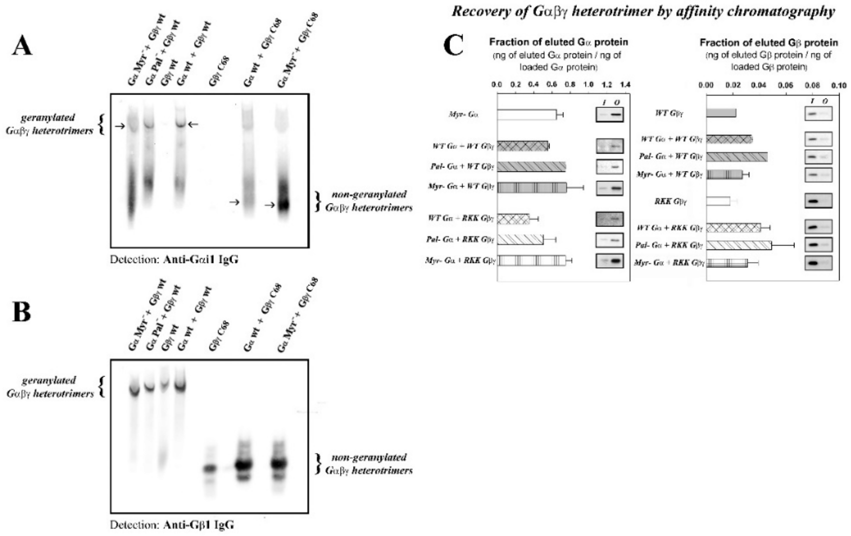

Figure S6

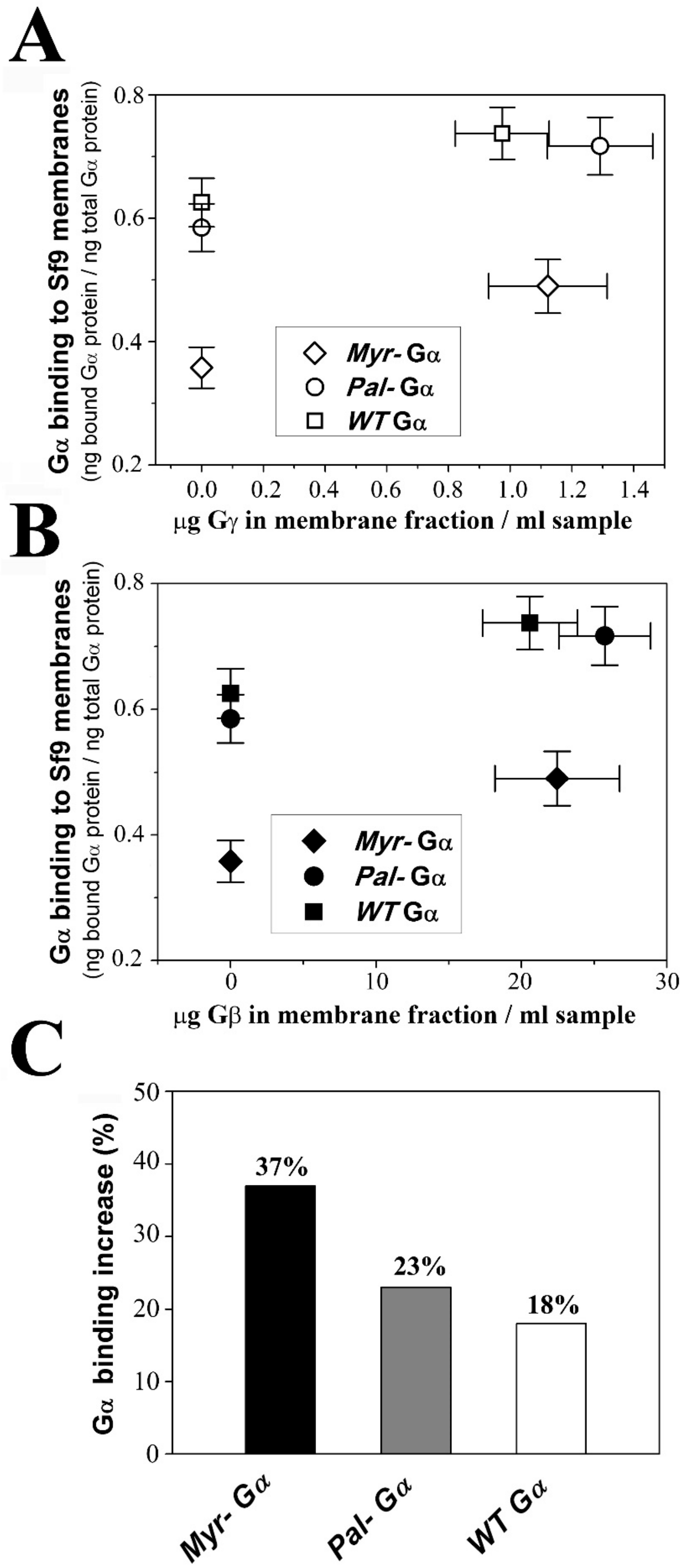
A
0.8
0.6
0.4
0.2
0.0 0.2 0.4 0.6 0.8 1.0 1.2 1.4
Gα binding to Sf9 membranes
(ng bound Gα protein / ng total Gα protein)
µg Gγ in membrane fraction / ml sample
Myr- Gα
Pal- Gα
WT Gα
B
0.8
0.6
0.4
0.2
0 10 20 30
Gα binding to Sf9 membranes
(ng bound Gα protein / ng total Gα protein)
µg Gβ in membrane fraction / ml sample
Myr- Gα
Pal- Gα
WT Gα
C
50
40
30
20
10
0
Gα binding increase (%)
37%
23%
18%
Myr- Gα
Pal- Gα
WT Gα

Figure S7

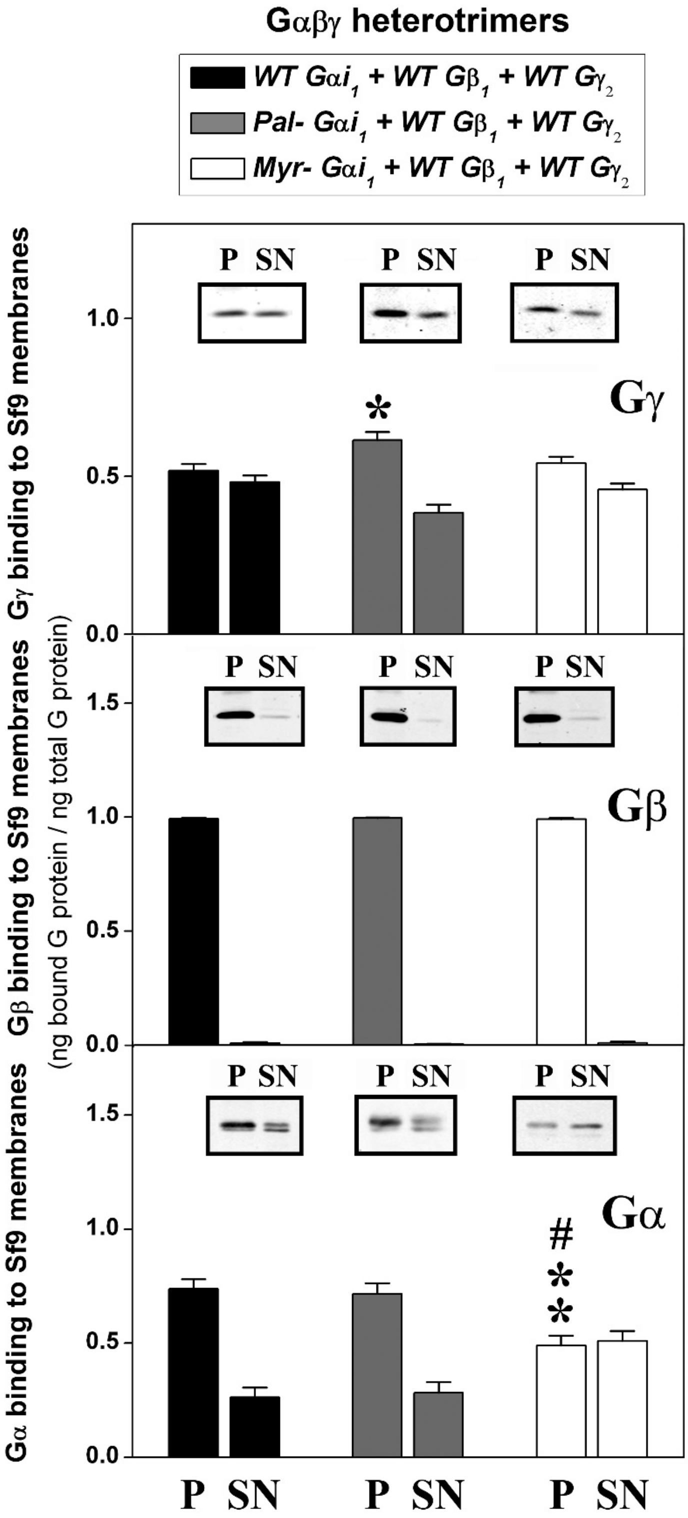
Gαβγ heterotrimers
WT Gαi1 + WT Gβ1 + WT Gγ2
Pal- Gαi1 + WT Gβ1 + WT Gγ2
Myr- Gαi1 + WT Gβ1 + WT Gγ2
Gγ binding to Sf9 membranes
Gβ binding to Sf9 membranes
Gα binding to Sf9 membranes
(ng bound G protein / ng total G protein)
Gγ
Gβ
Gα
P SN
P SN
P SN
0.0
0.5
1.0
1.5
*
#

Figure S8

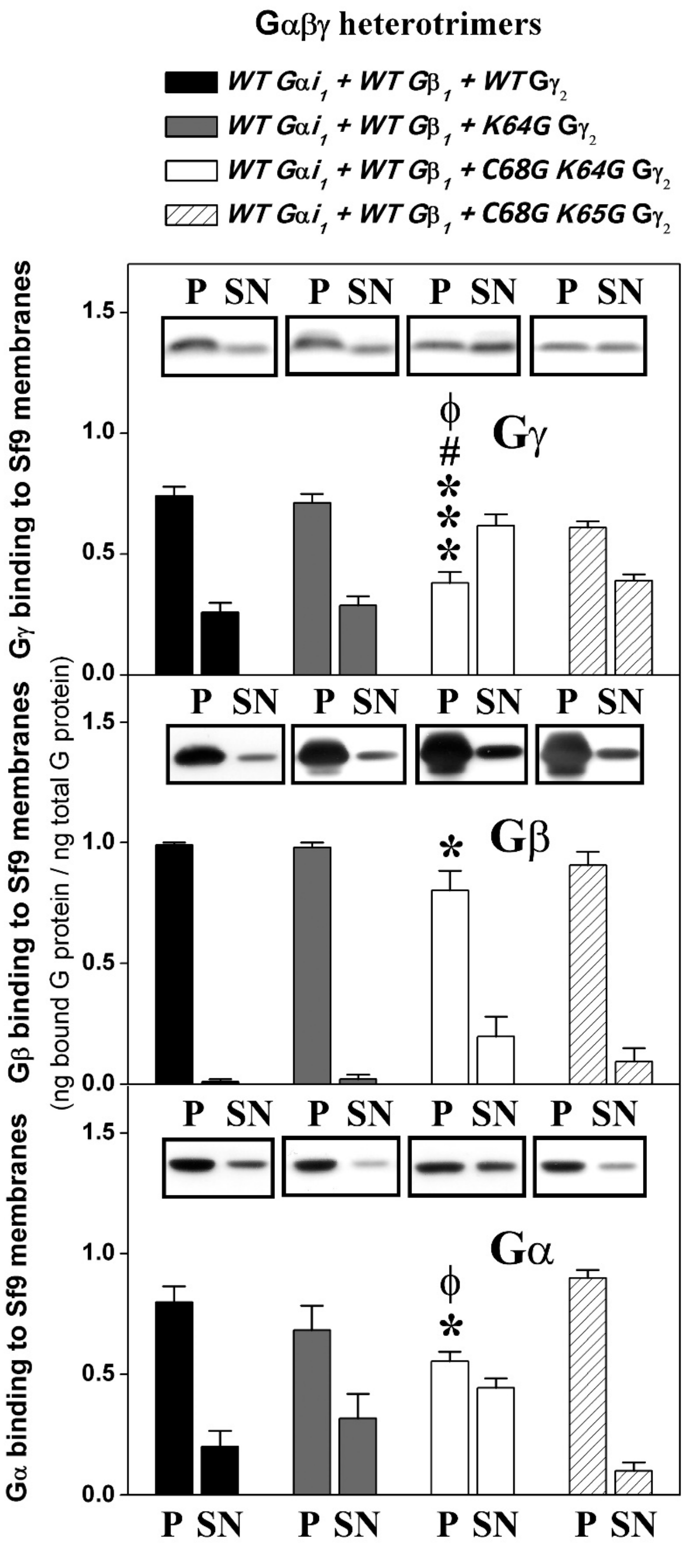

Gαβγ heterotrimers
WT Gαi1 + WT Gβ1 + WT Gγ2
WT Gαi1 + WT Gβ1 + K64G Gγ2
WT Gαi1 + WT Gβ1 + C68G K64G Gγ2
WT Gαi1 + WT Gβ1 + C68G K65G Gγ2
Gγ binding to Sf9 membranes
Gβ binding to Sf9 membranes
Gα binding to Sf9 membranes
(ng bound G protein / ng total G protein)
Gγ
Gβ
Gα
P SN
0.0
0.5
1.0
1.5

Figure S9

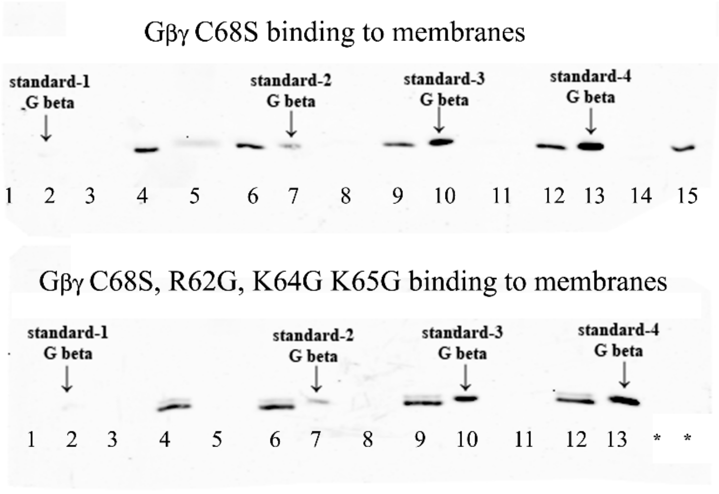

1 Mw marker
2 Gβ standard 2.62 ng
3 PC pellet + G dimer
4 PC supernatant + G dimer
5 PC:PE (1:1) pellet
6 PC:PE supernatant
7 Gβ standard 5.24 ng
8 PC:PS (8:2) pellet
9 PC:PS supernatant
10 Gβ standard 10.48 ng
11PC:PE:PS (2:2:1) pellet
12 PC:PE:PS supernatant
13 Gβ standard 17.46 ng
14 PC:PE:SM:Cho (1:1:1:1) pellet*
15 PC:PE:SM:Cho supernatant*
***Not loaded for lower blot**

Figure S10A

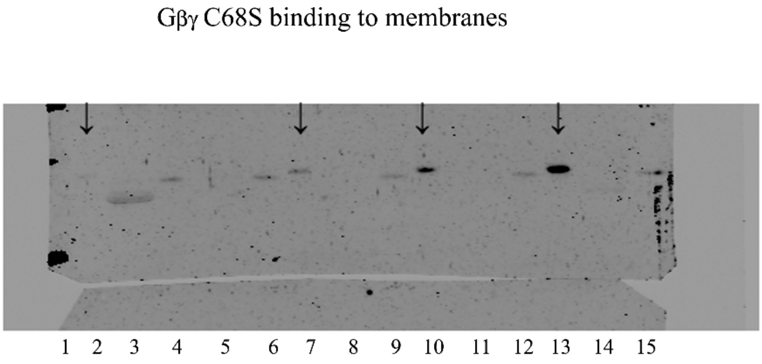

1 Mw marker
2 Gβ standard 2.62 ng
3 PC pellet + G dimer
4 PC supernatant + G dimer
5 PC:PE (1:1) pellet
6 PC:PE supernatant
7 Gβ standard 5.24 ng
8 PC:PS (8:2) pellet
9 PC:PS supernatant
10 Gβ standard 10.48 ng
11 PC:PE:PS (2:2:1) pellet
12 PC:PE:PS supernatant
13 Gβ standard 17.46 ng
14 PC:PE:SM:Cho (1:1:1:1) pellet
15 PC:PE:SM:Cho supernatant

Figure S10B

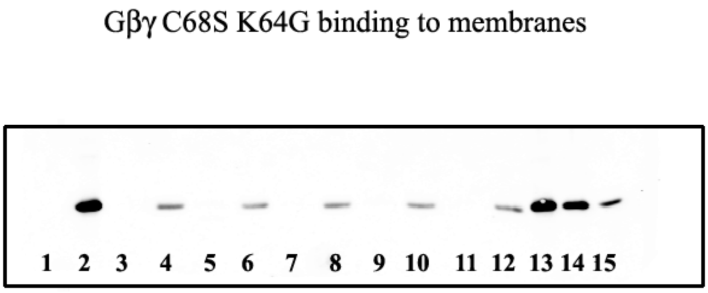

1 Mw marker
2 Gβ standard 94.32 ng
3 PC pellet + G dimer
4 PC supernatant + G dimer
5 PC:PE (1:1) pellet
6 PC:PE supernatant
7 PC:PS (8:2) pellet
8 PC:PS supernatant
9 PC:PE:PS (2:2:1) pellet
10 PC:PE:PS supernatant
11 PC:PE:SM:Cho (1:1:1:1) pellet
12 PC:PE:SM:Cho supernatant
13 Gβ standard 62.88 ng
14 Gβ standard 31.44 ng
15 Gβ standard 15.72 ng

Figure S10C

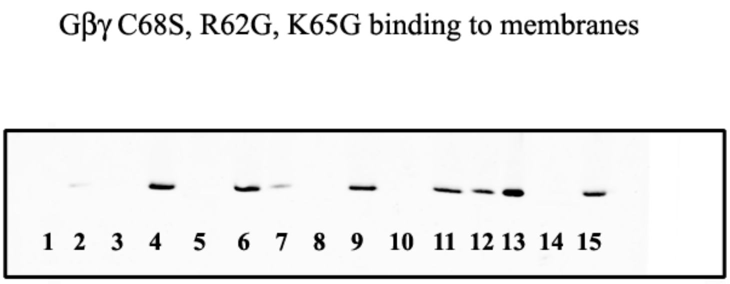

1 Mw marker
2 Gβ standard 3 ng
3 PC pellet + G dimer
4 PC supernatant + G dimer
5 PC:PE (1:1) pellet
6 PC:PE supernatant
7 Gβ standard 6 ng
8 PC:PS (8:2) pellet
9 PC:PS supernatant
10 PC:PE:PS (2:2:1) pellet
11 PC:PE:PS supernatant
12 Gβ standard 12 ng
13 Gβ standard 24 ng
14 PC:PE:SM:Cho (1:1:1:1) pellet
15 PC:PE:SM:Cho supernatant

Figure S10D

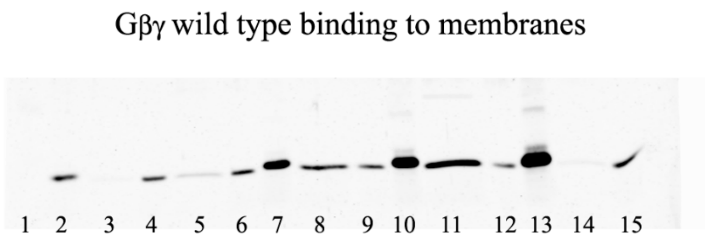

1 Mw marker
2 Gβ standard 2.62 ng
3 PC pellet + G dimer
4 PC supernatant + G dimer
5 PC:PS (8:2) pellet
6 PC:PS supernatant
7 Gβ standard 5.24 ng
8 PC:PE(1:1) pellet
9 PC:PE supernatant
10 Gβ standard 10.48 ng
11 PC:PE:PS (2:2:1) pellet
12 PC:PE:PS supernatant
13 Gβ standard 17.46 ng
14 PC:PE:SM:Cho (1:1:1:1) pell[illegible]
15 PC:PE:SM:Cho supernatant

Figure S10E

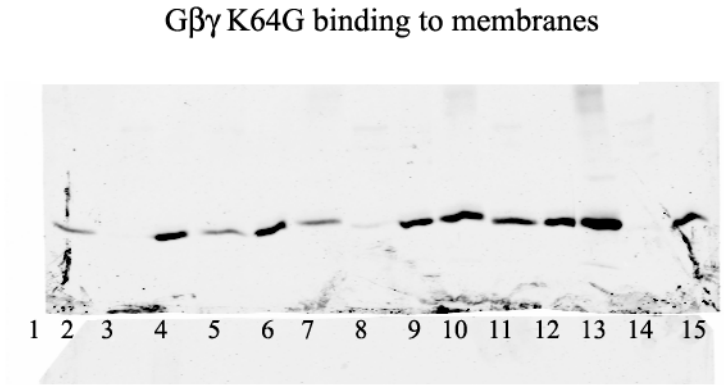

1 Mw marker
2 Gβ standard 2.6 ng
3 PC pellet + G dimer
4 PC supernatant + G dimer
5 PC:PE (1:1) pellet
6 PC:PE supernatant
7 Gβ standard 5.2 ng
8 PC:PS (8:2) pellet
9 PC:PS supernatant
10 Gβ standard 10.4 ng
11 PC:PE:PS (2:2:1) pellet
12 PC:PE:PS supernatant
13 Gβ standard 20.8 ng
14 PC:PE:SM:Cho (1:1:1:1) pellet
15 PC:PE:SM:Cho supernatant

Figure S10F

Gβγ K64G, K65G binding to membranes

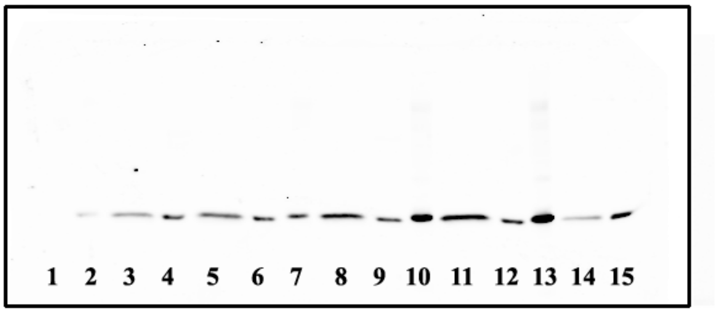

1 Mw marker
2 Gβ standard 15.72 ng
3 PC pellet + G dimer
4 PC supernatant + G dimer
5 PC:PE (1:1) pellet
6 PC:PE supernatant
7 Gβ standard 31.44 ng
8 PC:PS (8:2) pellet
9 PC:PS supernatant
10 Gβ standard 62.88 ng
11 PC:PE:PS (2:2:1) pellet
12 PC:PE:PS supernatant
13 Gβ standard 94.32 ng
14 PC:PE:SM:Cho (1:1:1:1) pellet
15 PC:PE:SM:Cho supernatant

Figure S10G

Gβγ K65G binding to membranes

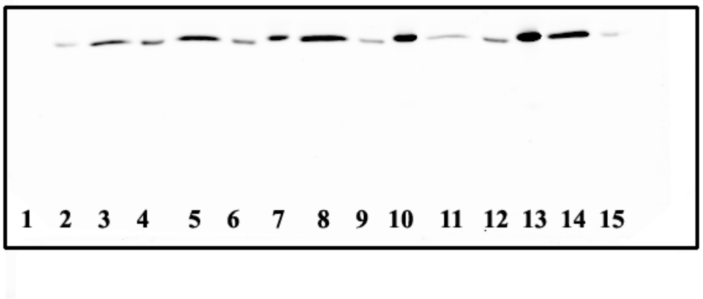

1 Mw marker
2 Gβ standard 15.72 ng
3 PC pellet + G dimer
4 PC supernatant + G dimer
5 PC:PE (1:1) pellet
6 PC:PE supernatant
7 Gβ standard 31.44 ng
8 PC:PS (8:2) pellet
9 PC:PS supernatant
10 Gβ standard 62.88 ng
11 PC:PE:SM:Cho (1:1:1:1) pellet
12 PC:PE:SM:Cho supernatant
13 Gβ standard 94.32 ng
14 PC:PE:PS (2:2:1) pellet
15 PC:PE:PS supernatant

Figure S10H

Gβγ R62G binding to membranes

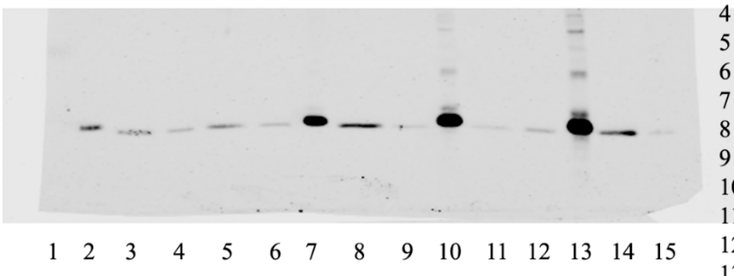

1 Mw marker
2 Gβ standard 2.62 ng
3 PC pellet + G dimer
4 PC supernatant + G dimer
5 PC:PE (1:1) pellet
6 PC:PE supernatant
7 Gβ standard 5.24 ng
8 PC:PS (8:2) pellet
9 PC:PS supernatant
10 Gβ standard 10.48 ng
11 PC:PE:SM:Cho (1:1:1:1) pellet
12 PC:PE:SM:Cho supernatant
13 Gβ standard 17.46 ng
14 PC:PE:PS (2:2:1) pellet
15 PC:PE:PS supernatant

Figure S10I

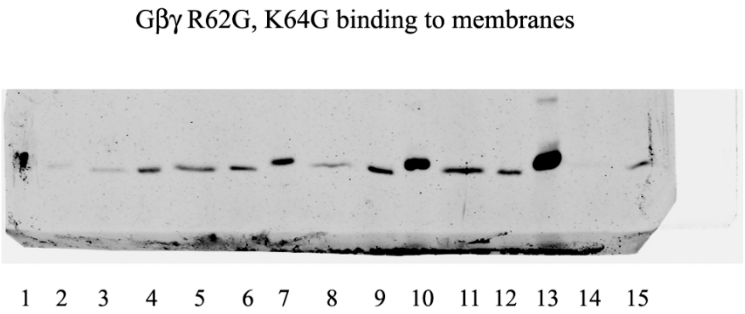

1 Mw marker
2 Gβ standard 2.62 ng
3 PC pellet + G dimer
4 PC supernatant + G dimer
5 PC:PE (1:1) pellet
6 PC:PE supernatant
7 Gβ standard 5.24 ng
8 PC:PS (8:2) pellet
9 PC:PS supernatant
10 Gβ standard 10.48 ng
11 PC:PE:PS (2:2:1) pellet
12 PC:PE:PS supernatant
13 Gβ standard 17.46 ng
14 PC:PE:SM:Cho (1:1:1:1) pellet
15 PC:PE:SM:Cho supernatant

Figure S10J

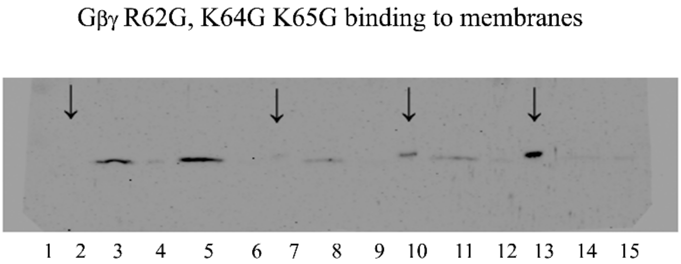

1 Mw marker
2 Gβ standard 2.62 ng
3 PC pellet + G dimer
4 PC supernatant + G dimer
5 PC:PE (1:1) pellet
6 PC:PE supernatant
7 Gβ standard 5.24 ng
8 PC:PS (8:2) pellet
9 PC:PS supernatant
10 Gβ standard 10.48 ng
11 PC:PE:PS (2:2:1) pellet
12 PC:PE:PS supernatant
13 Gβ standard 17.46 ng
14 PC:PE:SM:Cho (1:1:1:1) pellet
15 PC:PE:SM:Cho supernatant

Figure S10K

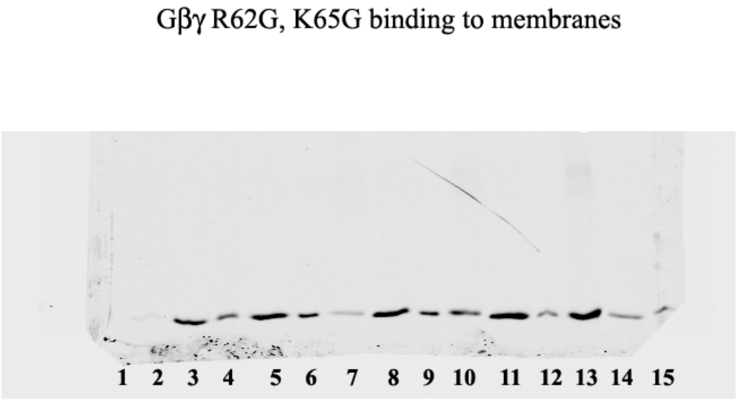

1 Mw marker
2 Gβ standard 2.62 ng
3 PC pellet + G dimer
4 PC supernatant + G dimer
5 PC:PE (1:1) pellet
6 PC:PE supernatant
7 Gβ standard 5.24 ng
8 PC:PS (8:2) pellet
9 PC:PS supernatant
10 Gβ standard 10.48 ng
11 PC:PE:PS (2:2:1) pellet
12 PC:PE:PS supernatant
13 Gβ standard 17.46 ng
14 PC:PE:SM:Cho (1:1:1:1) pellet
15 PC:PE:SM:Cho supernatant

Figure S10L

Gαβγ: Myr- Gα / Gβγ RKK binding to membranes

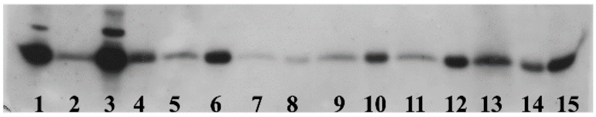

1 Gβ standard 40 ng
2 PC pellet
3 Gβ standard 80 ng
4 PC supernatant
5 PC:PE (1:1) pellet
6 PC:PE (1:1) supernatant
7 PC:PS (8:2) pellet
8 PC:PS (8:2) supernatant
9 PC:PS pellet
10 PC:PS supernatant
11 PC:PE:PS (2:2:1) pellet
12 PC:PE:PS (2:2:1) supernatant
13 PC:PE:SM:Cho (1:1:1:1) pellet
14 PC:PE:SM:Cho (1:1:1:1) supernatant
15 Mw marker+Gβ standard 20 ng

Figure S10M

Gαβγ: Myr- Gα / Gβγ WT binding to membranes

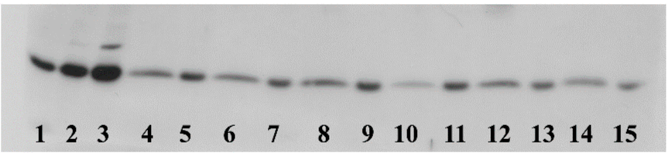

1 Gβ standard 80 ng+Mw marker
2 Gβ standard 40 ng
3 Gβ standard 20 ng
4 PC pellet
5 PC supernatant
6 PC:PE (1:1) pellet
7 PC:PE (1:1) supernatant
8 PC:PS (8:2) pellet
9 PC:PE:SM:Cho (1:1:1:1) supernatant
10 PC:PE:SM:Cho (1:1:1:1) pellet
11 PC:PS (8:2) supernatant
12 PC:PS (8:2) pellet
13 PC:PS (8:2) supernatant
14 PC:PE:PS (2:2:1) pellet
15 PC:PE:PS (2:2:1) supernatant

Figure S10N

Gαβγ: Pal- Gα / Gβγ RKK binding to membranes

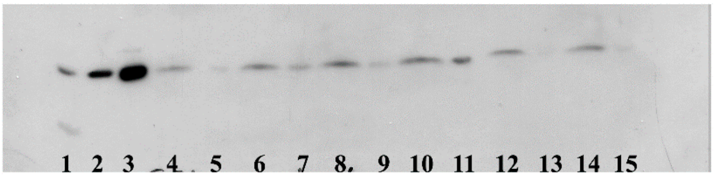

1 Mw marker+Gβ standard 7.5 ng
2 Gβ standard 15 ng
3 Gβ standard 30 ng
4 PC pellet
5 PC supernatant
6 PC:PS (8:2) pellet
7 PC:PS (8:2) supernatant
8 PC:PS (8:2) pellet
9 PC:PS (8:2) supernatant
10 PC:PE (1:1) pellet
11 PC:PE (1:1) supernatant
12 PC:PE:SM:Cho (1:1:1:1) pellet
13 PC:PE:SM:Cho (1:1:1:1) supernat
14 PC:PE:PS (2:2:1) pellet
15 PC:PE:PS (2:2:1) supernatant

Figure S10O

Gαβγ: Pal- Gα / Gβγ WT binding to membranes

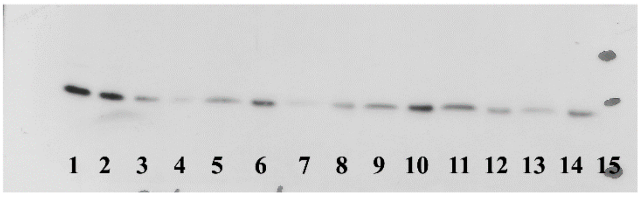

1 Gβ standard 80 ng
2 Gβ standard 40 ng
3 Gβ standard 20 ng
4 Gβ standard 10 ng
5 PC pellet
6 PC supernatant
7 PC:PE (1:1) pellet
8 PC:PE (1:1) supernatant
9 PC:PS (8:2) pellet
10 PC:PS (8:2) supernatant
11 PC:PE:PS (2:2:1) pellet
12 PC:PE:PS (2:2:1) supernatant
13 PC:PE:SM:Cho (1:1:1:1) pellet
14 PC:PE:SM:Cho (1:1:1:1) supernatant
15 Mw marker

Figure S10P

Gαβγ: Pal+ Gα / Gβγ RKK binding to membranes

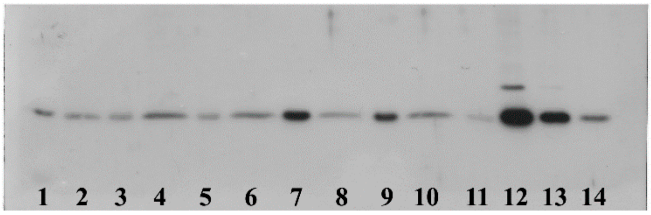

1 Mw marker+Gβ standard 3.75 ng
2 PC pellet
3 PC supernatant
4 PC:PE (1:1) pellet
5 PC:PE (1:1) supernatant
6 PC:PS (8:2) pellet
7 PC:PS (8:2) supernatant
8 PC:PE:SM:Cho (1:1:1:1) pellet
9 PC:PE:SM:Cho (1:1:1:1) supernatant
10 PC:PE:PS (2:2:1) pellet
11 PC:PE:PS (2:2:1) supernatant
12 Gβ standard 30 ng
13 Gβ standard 15 ng
14 Gβ standard 7.5 ng

Figure S10Q

Gαβγ: Pal+ Gα / Gβγ WT binding to membranes

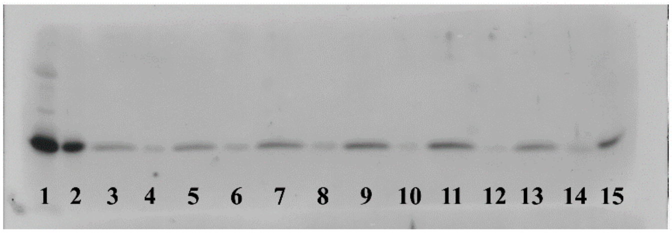

1 Mw marker+G prot standard 80 ng
2 G prot standard 40 ng
3 PC:PS (8:2) pellet Gαβγ
4 PC:PS (8:2) supernatant Gαβγ
5 PC:PS (8:2) pellet
6 PC:PS (8:2) supernatant
7 PC pellet
8 PC supernatant
9 PC:PE (1:1) pellet
10 PC:PE (1:1) supernatant
11 PC:PE:PS (2:2:1) pellet
12 PC:PE:PS (2:2:1) supernatant
13 PC:PE:SM:Cho (1:1:1:1) pellet
14 PC:PE:SM:Cho (1:1:1:1) supernatant
15 G prot standard 20 ng

Figure S10R
